# Interfacial States in Au/Reduced TiO_2_ Plasmonic Photocatalysts Quench Hot-Carrier Photoactivity

**DOI:** 10.1021/acs.jpcc.3c04176

**Published:** 2023-08-07

**Authors:** Olivier Henrotte, Štěpán Kment, Alberto Naldoni

**Affiliations:** † Czech Advanced Technology and Research Institute, Regional Centre of Advanced Technologies and Materials Department, Palacký University Olomouc, Šlechtitelů 27, Olomouc 78371, Czech Republic; ‡ CEET, Nanotechnology Centre, VŠB-Technical University of Ostrava, 17. Listopadu 2172/15, Ostrava-Poruba 708 00, Czech Republic; § Department of Chemistry and NIS Centre, University of Turin, Turin 10125, Italy

## Abstract

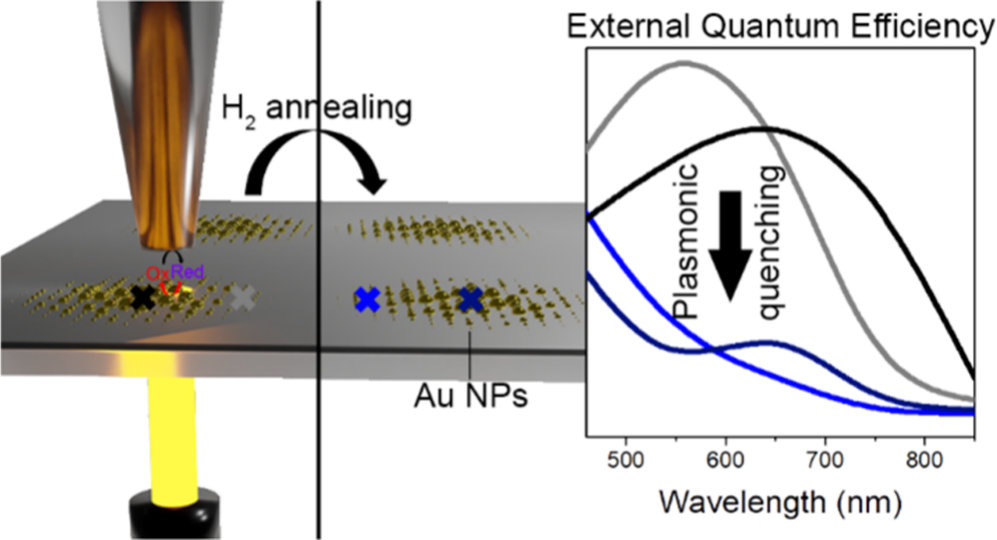

Understanding the interface of plasmonic nanostructures is essential for improving the performance of photocatalysts. Surface defects in semiconductors modify the dynamics of charge carriers, which are not well understood yet. Here, we take advantage of scanning photoelectrochemical microscopy (SPECM) as a fast and effective tool for detecting the impact of surface defects on the photoactivity of plasmonic hybrid nanostructures. We evidenced a significant photoactivity activation of TiO_2_ ultrathin films under visible light upon mild reduction treatment. Through Au nanoparticle (NP) arrays deposited on different reduced TiO_2_ films, the plasmonic photoactivity mapping revealed the effect of interfacial defects on hot charge carriers, which quenched the plasmonic activity by (i) increasing the recombination rate between hot charge carriers and (ii) leaking electrons (injected and generated in TiO_2_) into the Au NPs. Our results show that the catalyst’s photoactivity depends on the concentration of surface defects and the population distribution of Au NPs. The present study unlocks the fast and simple detection of the surface engineering effect on the photocatalytic activity of plasmonic semiconductor systems.

## Introduction

The production of chemicals through renewable and clean processes has grown into the holy grail road over the last century due to the alarming consumption rate of finite resources (fossil fuels) and the technological prowess unlocking the inextinguishable energy source handlings (e.g., solar and wind energy).^1^ Photodevices received particular attention because of the massive amount of energy provided by the daily sunlight irradiating Earth.^2^ However, there are still many obstacles to producing mature systems reaching industrials. Indeed, solar-to-chemical devices need to absorb sunlight (mainly visible light) efficiently while generating sufficiently energetic charge carriers to interact with molecules surrounding the devices.^3^ To address these stringent requirements, one possible route consists in using hybrid metal–semiconductor nanostructures combining the former’s exceptional optical properties and the possibility of forming rectifying junctions.^3−5^ Furthermore, the fabrication of properly designed hybrid nanosystems requires considerable effort in understanding both parts and, even more, their interfaces. For example, the optical properties of the nanostructures are linked directly to their size, shape, composition, and embedding media, while the generated charge carrier energy, lifetime, and availability for redox processes rely on their surface states (e.g., crystallinity, defects, etc..) and the metal–semiconductor interface as well as with the surrounding media status.^6−8^

One of the most studied hybrid nanosystems consists of plasmonic gold nanoparticles (NPs) deposited on TiO_2_ (i.e., cheap, abundant, stable, and environmentally friendly semiconductor) because of the numerous applications in photodevices. As such, extensive research efforts were devoted to the understanding of underlying mechanisms behind the hot charge carrier generation through Au surface plasmon excitation and their subsequent separation and utilization. To this end, Au NPs are coupled with TiO_2_, forming a junction consisting of a Schottky barrier (ϕ_B_) with energy specific to the system (e.g., Au-TiO_2_ ϕ_B_ ≈ 1.1 eV)^9^ that hot charge carriers need to overcome to be injected and stabilized into the semiconductor conduction band (CB). Interestingly, the surface state of TiO_2_ directly affects the photoactivity of Au-TiO_2_ nanostructures.

Induced defects such as oxygen vacancies (V_O_s) and Ti^3+^ states are well-known because of their simple formation process (e.g., thermal treatment in a reducing atmosphere) while significantly affecting the photocatalyst properties.^6−8,10−12^ For instance, Chen et al. showed the narrowing of the TiO_2_ optical band gap from 3.3 to 1.54 eV through lattice disorder, inducing defect states (DSs) stabilized by hydrogen atoms.^13^ The V_O_s, from which Ti^3+^ states arise, introduced by thermal treatment under H_2_, correspond to defect levels 0.7–1 eV below the CB.^14^ While the DSs enhance the optical properties of TiO_2_, the photocatalytic activity of TiO_2_ for specific reactions (e.g., H^+^/H_2_ redox potential) showed lower efficiency due to the DS energy level below the redox potential.^14,15^ This demonstrates the need for a better understanding and controlled engineering of DSs in photocatalytic materials.

The critical role of the interface between the metal and semiconductor on the photocatalytic activity of the nanosystem was regularly demonstrated. The adoption of n- or p-type semiconductors dictates the hot charge carrier flow in the hybrid nanosystem.^16−18^ Additionally, the interfacial state significantly modifies the material’s photoactivity. In the case of Au-TiO_2_, Lin et al. reported that V_O_s in TiO_2–*x*_ enabled an enhancement in the localized surface plasmon resonance (LSPR) at 2.3 eV while diminishing the ϕ_B_ height by 5 mV compared to pristine TiO_2_.^11^ Li et al. reported that interfacial defects from reduced TiO_2_ acted as intragap states trapping hot electrons generated from plasmon excitation, allowing them a backward path and consequently decreasing their separation.^12^ Similarly, Naldoni et al. demonstrated a one-order-of-magnitude decrease in the H_2_ production for Au-black TiO_2_ compared to Au-pristine TiO_2_.^15^

To date, there has not been an established consensus on how such defects affect photocatalytic processes, which remains a highly debated issue. This is due in part to the difficult DS detection and its associated comprehension. A large panel of detection methods (e.g., X-ray diffraction (XRD), X-ray photoelectron spectroscopy (XPS), Raman spectroscopy, photoluminescence (PL) spectroscopy, high-resolution transmission electron microscopy (HR-TEM), and electron paramagnetic resonance (EPR)) has been employed to detect V_O_/Ti^3+^ states in TiO_2_.^11,19−22^ While those techniques are efficient in identifying V_O_s in these nanostructures, they require particular conditions, which frequently differ from those used in photocatalytic experiments. Moreover, defect detection becomes harder in ultrathin films treated under mild conditions, i.e. inducing a lower concentration of defects, rendering blind most of the available detection techniques.

**Figure 1:**
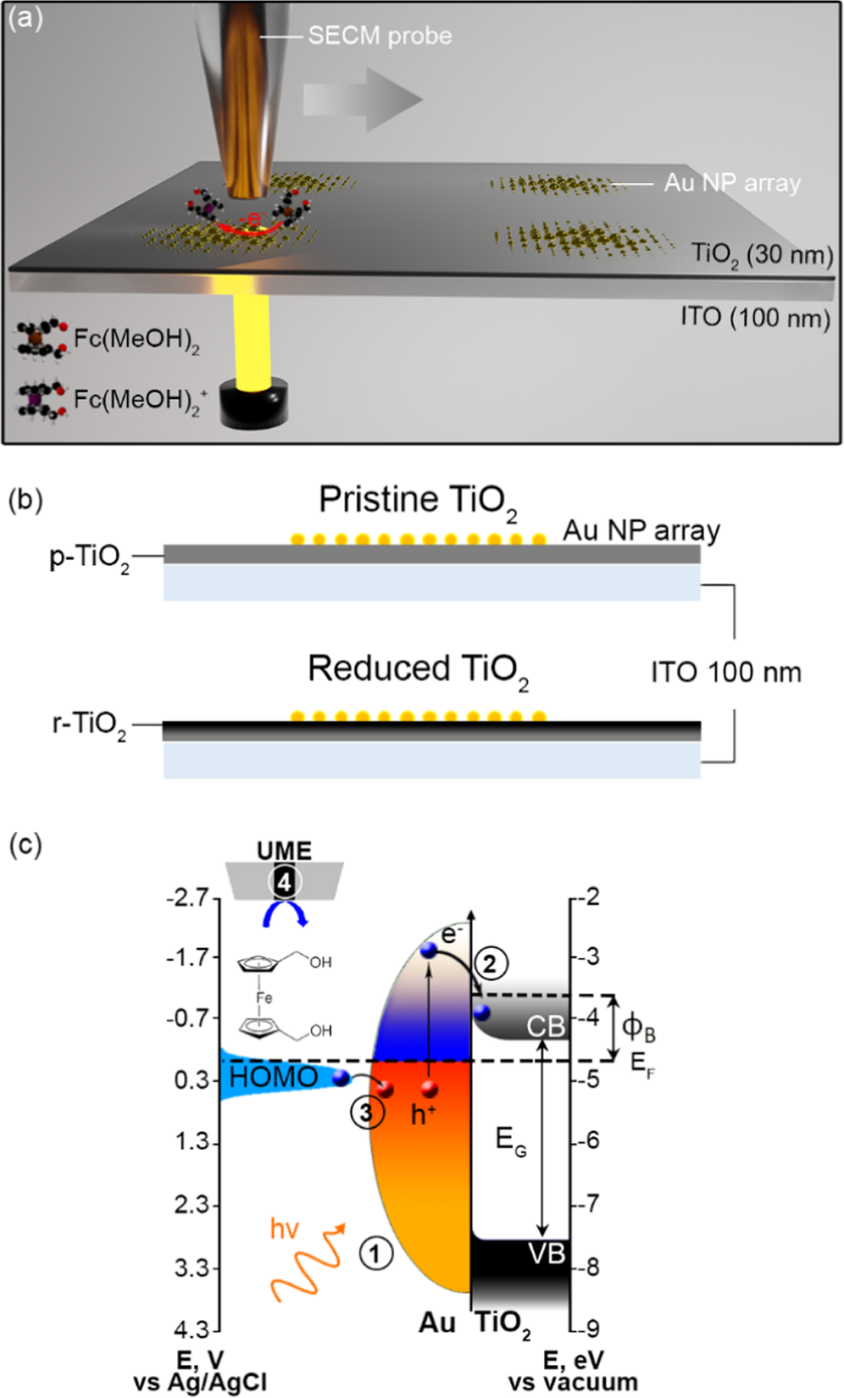
(a) Scheme depicting the SPECM measurements performed on Au-TiO_2_ samples. (b) Schematics of the Au NP array deposited on the pristine and reduced TiO_2_–ITO underlayers. (c) Illustration of the mechanism underlying the plasmonic reactivity quantification for Au NPs on TiO_2_ photocatalysts.

Herein, we demonstrate, through scanning photoelectrochemical microscopy (SPECM), the possibility of studying the impact of induced defects generated via mild treatments over ultrathin films (∼30 nm) on the plasmon-driven photooxidation of chemicals from a model plasmonic photocatalyst; i.e., Au NP arrays on TiO_2_ films (Figure 1a). We chose Au-TiO_2_ photocatalysts because of their well-referenced plasmonic and photochemical properties.^16,23−28^ We recently reported that SPECM, combined with other scanning probe microscopy techniques, enables the local spatial detection and quantification of molecular products during plasmonic hot-carrier-driven photocatalytic reactions.^29^ To do so, we investigated different ultrathin films of TiO_2_ (pristine and reduced) by SPECM correlating the applied reduction process to the external quantum efficiency (EQE) under sub-band-gap excitation (2.72, 2.34, 1.88, 1.67, and 1.45 eV). Au NP arrays were carefully designed to present a similar population distribution on the different TiO_2_ films. Further, SPECM enabled the photoactivity mapping of the different Au NP arrays and their corresponding EQE depending on the Au NP population distribution and the applied reduction process. Interestingly, we observed significant quenching of the plasmonic activity, which was directly related to the Au NP population distribution and the quantity of DSs in TiO_2_. The wavelength-dependent charge carrier generation and recombination processes were significantly altered by the applied reduction process. Our results offer a glimpse of the impact of interfacial defects on (i) the hot charge carrier recombination processes and (ii) the plasmonic quenching of the Au NPs from charge carriers generated into TiO_2_. As evidenced by our results, such hybrid plasmonic nanosystems are extremely complex and we expect our findings to refine the understanding of interfacial defects in hybrid nanostructures.^9,11,12,15,30^

## Methods

### Au NPs on TiO_2_ Ultrathin Film Fabrication

The Au-TiO_2_ nanostructures were fabricated on indium tin oxide (ITO) coated glass slides with a size of 25 × 25 mm^2^ (Ossila, U.K.). The substrates were cleaned using ultrasonication for 5 min each in acetone, ethanol, and then deionized water prior to use. Plasma-assisted atomic layer deposition (ALD, Ultratech/CambridgeNanoTech Fiji 200) using tetrakis(dimethylamido)titanium as a precursor (99% purity, Strem) at 250 °C was adopted for the deposition of a 30 nm TiO_2_ film. One ALD cycle consisted of 0.1 s of precursor exposure, 5 s of purge, 20 s of O_2_ plasma exposure (300 W), and 5 s of purge of reaction products. Then, to guarantee the anatase phase formation, the TiO_2_ ultrathin layers were annealed in air at 450 °C (heating rate of 2 °C/min) for 2 h. The Au NP arrays were produced by dewetting in air at 400 °C (heating rate of 2 °C/min) for 1 h of a sputtered Au film (10 nm) through a patterned mask (Micronlaser Technology) consisting of 16 × 16 squares (100 × 100 μm^2^; 400 μm interdistance). To obtain reduced TiO_2_, the TiO_2_ layers were further annealed under a gas mixture of Ar/H_2_ (95%/5%) at different temperatures (250 and 350 °C) with a heating rate of 2 °C/min for 1 h. The same process was applied to fabricate the TiO_2_ films on the Si wafer employed for physical characterizations.

### Physical Characterization

The nanomaterials’ morphology was investigated by a field emission scanning electron microscope (FE-SEM, Hitachi SU 6600, Japan). ImageJ was employed to obtain the Au NP size distribution through a contrast filter. The calculated diameter of the Au NPs (approximated as a circumference) was retrieved thanks to the NP areas obtained by ImageJ. XPS analysis was performed on a Nexsa G2 (Thermo Fisher Scientific) with an Al Kα source (photon energy of 1486.7 eV; spot size of 100 μm). The obtained data were evaluated by using Avantage software and CasaXPS. High-resolution spectra were scaled using the adventitious carbon peak as a reference. Raman spectra were collected using a DXR Raman spectrometer (Thermo Scientific, Massachusetts) operating at 633 nm and 4 mW. The PL spectroscopy measurements were performed on an FLS980 fluorescence spectrometer (Edinburgh Instruments) with double monochromators on both excitation and emission sides, equipped with an R928P photomultiplier in a thermoelectrically cooled housing (Hamamatsu Photonics), with a 450 W xenon arc lamp as the excitation source. Spectral correction curves were provided by Edinburgh Instruments.

### Scanning Spectrophotometer Microscopy

A spectrophotometer coupled with the positioning system of the scanning electrochemical microscope enabled local investigation of the samples’ optical properties (HEKA Gmbh, Lambrecht, Germany). A white lamp was focused through an objective (LUCPLFLN 40×, NA = 0.6, Olympus, Japan) below the sample and aligned with a glass capillary (100 μm radius). An optical fiber (100 μm radius, NA = 0.22, Thorlabs) was connected to the glass capillary, transmitting the light to the spectrophotometer. The light beam radius was ∼5 μm, and the lamp used was a Lambda LS Xenon Arc bulb (wavelength from 330 to 780 nm, Sutter instruments). The measured spectra were extracted from the localized transmission spectra at different XY positions of the sample (every 20 μm). The values obtained from the spectra at the different XY positions correspond to −log(*T*), where , with *I* and *I*_0_ being the transmitted light through the measured sample and the bare substrate (without Au NPs), respectively. The optical properties at shorter and longer wavelengths (<500 and >700 nm) were bounded by the white lamp’s limitation in wavelength and light intensities.

### Scanning Photoelectrochemical Microscopy

The SPECM employed monochromatic LED light with a beam radius similar to that of the scanning spectrophotometer microscope (SSM) configuration to locally irradiate the sample (from below) and drive the photochemical reactions. The SPECM experiments were performed using an ELP 3 SPECM-FL (HEKA Elektronik GmbH, Lambrecht, Germany) including a PG 618 USB bipotentiostat (HEKA Elektronik GmbH, Lambrecht, Germany) and fiber-coupled LED lamps with different wavelengths: 455 ± 7, 530 ± 15, 660 ± 9, 740 ± 11, and 850 ± 15 nm (Thorlabs). The experiments were conducted in 0.1 M KCl and 1 mM Fc(MeOH)_2_ (ferrocene dimethanol, FeC_12_H_14_O_2_) under air and at room temperature. Chemicals were purchased from Sigma-Aldrich with the highest purity and used without further purification. Milli-Q water was used for the preparation of all aqueous solutions. The SPECM setup is composed of a four-electrode cell: two working electrodes (the substrate: WE1, not connected in this study, and the Pt ultra-microelectrode (UME): WE2), the reference electrode (Ag/AgCl wire), and the counter electrode (Pt wire). The UME (HEKA Elektronik GmbH, Lambrecht, Germany) used in this work had *r*_T_ = 5 μm and RG = 5. The probe moves near the studied sample measuring locally the concentration evolution of the species in solution (depending on the WE2 applied bias). The alignment of the light beam and probe was verified between each measurement. The XY stage enabled the scanning of the samples with the probe placed at a constant distance (*d*), thanks to the Z stage. At the substrate, the redox mediator is oxidized from the hot holes as follows
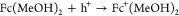
1The concentrations of both species (reduced and oxidized) near the beam position are locally modified. The probe detects this modification by electrochemically reducing back (substrate generation–tip collection, SG-TC mode) the species photoproduced by the substrate
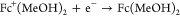
2The probe current (*I*_T_) corresponds to a steady-state regime because the applied bias consists of a potential (*E*_Tip_) where the diffusion limits the probe current (0 V vs Ag/AgCl in KCl 0.1 M).^29^ This current is theoretically calculated by eq 3 in the bulk solution (*I*_T,∞_) and eq 4 close to the substrate (*I*_T,ins_) due to the hindering effect on the diffusion of both the substrate and the probe (equivalent to a negative feedback)^31,32^
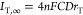
3
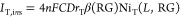
4where *n* is the number of electrons involved; *F* is the Faraday constant (96485.33 C/mol); *C* is the concentration in Red/Ox species; *D* is the diffusion coefficient of the Red/Ox species (7.8 × 10^–6^ cm^2^/s);^33^*r*_T_ is the probe active part radius; RG is the ratio between the probe insulating part radius and *r*_T_; β(RG) is the RG impact on the diffusion to the probe active area; and Ni_T_(*L*) is the hindering effect of the near-surface on the probe current. with *L* corresponding to the ratio between the probe–substrate distance (*d*) and *r*_T_. The SPECM map acquisition was performed with an XY scan. The probe scanned the sample at a constant speed (*v*_scan_). The obtained resolution of the SPECM measurements depends on r_T_, *d*, and the number of acquisitions and lines chosen during the scanning process. For instance, a 1 × 1 mm^2^ map corresponds to 1001 acquisitions (one acquisition every 1 μm) in X and 51 lines in Y (one line every 20 μm).

## Results and Discussion

Au NP arrays grown on ultrathin TiO_2_ films deposited over a charge collector (i.e., ITO) support (Figure 1b) previously served as a plasmonic system model to investigate the efficiency of hot holes generated upon illumination over the photooxidation of a redox probe.^29^ The mechanism underlying charge separation and molecular detection consists of the following steps (Figure 1c). (1) The hot carriers are generated in the Au NPs excited with visible light. (2) The hot electrons with sufficient energy to overcome the Schottky barrier (ϕ_B_) forming at the metal–semiconductor interface are injected into the TiO_2_ CB, while the corresponding hot holes accumulate in the Au NPs. The hot electrons are driven away thanks to the conductive underlayer (ITO) and react/relax either in TiO_2_/ITO DSs/doping centers or through bipolar electrochemical behavior.^26,29^ (3) The hot holes with sufficient energy at the Au NPs–electrolyte interface oxidize the molecular species surrounding the Au NPs. (4) The UME detects the oxidized species by electrochemically reducing them back. Ferrocene dimethanol (FcDM), Fc(MeOH)_2_, was used as a redox probe because of its outer-sphere one electron transfer mechanism, ensuring that the surface adsorption step during the catalytic reaction is negligible, thus not affecting the reaction kinetics.^34^ The highest occupied molecular orbital (HOMO) energy level of FcDM matches the Au-TiO_2_ Fermi level (*E*_F_).^26,35^ Thus, the photooxidized molecules’ rate is considered as the maximum number of hot holes reacting per unit of time.

We used SPECM to detect *in situ* the effect of the plasmonic photocatalysts on the local concentration evolution of molecular species photoproduced in solution under a steady-state regime. SPECM has been recently adopted to distinguish the possible mechanisms involved in surface plasmon photocatalysis.^26,29,36−39^ Here, we studied the oxidation reaction taking place at the substrate thanks to the UME using the SG–TC mode, which consists of electrochemically reducing back the oxidized species. The UME current measured under both dark and light conditions gave the purely photocatalytic activity by computing the differential current (Δ*I* = *I*_light_*– I*_dark_).

### Visible Light Activity of TiO_2_ Ultrathin Films

We fabricated TiO_2_ ultrathin films (30 nm) deposited on ITO-coated glass through ALD. A mild reduction treatment under an Ar/H_2_ stream at different temperatures (see Methods) was applied to produce a series of samples with various amounts of induced defects (V_O_ and Ti^3+^ states), and thus of intraband gap electronic states, into the TiO_2_ films.^7^ The different TiO_2_ samples, namely, pristine (p-) and reduced (r-) TiO_2_, were investigated by SPECM to probe the formation of defects and their impact on the TiO_2_ photocatalytic activity.

We started by looking into the physicochemical properties of the prepared TiO_2_ samples to find how the reduction treatments modified their features.

Raman spectra of p-TiO_2_ and the other samples show typical peaks related to the anatase phase (Figure S1). The *E*_g_ peak (∼144 cm^–1^) is often used to determine the presence of defects in anatase TiO_2_: the peak is shifting^40,41^ or broadening^42^ when the V_O_ concentration increases. Figure S1 shows that the *E*_g_ peak of the different TiO_2_ deposited on the Si wafer (TiO_2_–Si) did not shift or broaden despite the reduction process. It was not possible to perform the measurements on the TiO_2_ samples grown on ITO as the signal from the latter shadowed the TiO_2_*E*_g_ peak region (Figure S2).

Interestingly, PL spectra also showed no significant changes between pristine and reduced TiO_2_ (Figure S3), in contrast with previous results from our group for TiO_2_ thin films (but with a thickness of ∼300 nm) or nanorods.^20,43^ The reason behind these results may be due to the very low concentration of defects created by the presently applied mild thermal treatment and the ultrathin thickness of the films.^10,44^

On the other hand, XPS is a powerful technique that allows probing the surface composition of reducible oxides like TiO_2_, and it has been shown to be an effective tool to identify the formation of Ti^3+^ species and oxygen deficiency in reduced TiO_2_.^42,45,46^ From the XPS survey spectra of the different investigated TiO_2_ films (Figure S4), the composition in Ti species looks similar for the different TiO_2_ films, while the decrease in the number of O species (p-TiO_2_ > r-TiO_2_ (250 °C) > r-TiO_2_ (350 °C)) followed the increase in the number of C species, indicating that reduction processes effectively occurred with possible carbon species hydrogenation at the film surface (Table S1). As expected for mildly reduced TiO_2_, no significant changes between p-TiO_2_ and r-TiO_2_ were observed in the high-resolution Ti 2p spectra (Figure S5).^47^ In contrast, a clear trend was observed upon comparison of the spectra in the O 1s region (Figure S6). Each spectrum could be deconvoluted into two components belonging to lattice oxygen in TiO_2_ (∼530.6 eV) and oxygen related to adventitious carbon species (∼532.5 eV).^48^ The latter signal area contribution (*C*%_Adv.C;_Table S2) to the overall O 1s line shape underwent a monotonic decrease upon TiO_2_ reduction, following the order p-TiO_2_ > r-TiO_2_ (250 °C) > r-TiO_2_ (350 °C) due to the reduction of the TiO_2_ surface with possible adventitious carbon species modification. However, reports from different reduced TiO_2_ nanostructures propose an opposite behavior; this latter peak increases and corresponds to the nonlattice/water adsorbed species, further related to an increase in surface V_O_s.^46,49^ Moreover, a recent article evidenced the frequent misinterpretation of adsorbed H_2_O on metal oxides as V_O_s in the material.^50^ As such, we do not believe that this observation is reliable enough to conclude the presence of surface vacancies, and thus intragap electronic states, in the presented ultrathin films.

**Figure 2:**
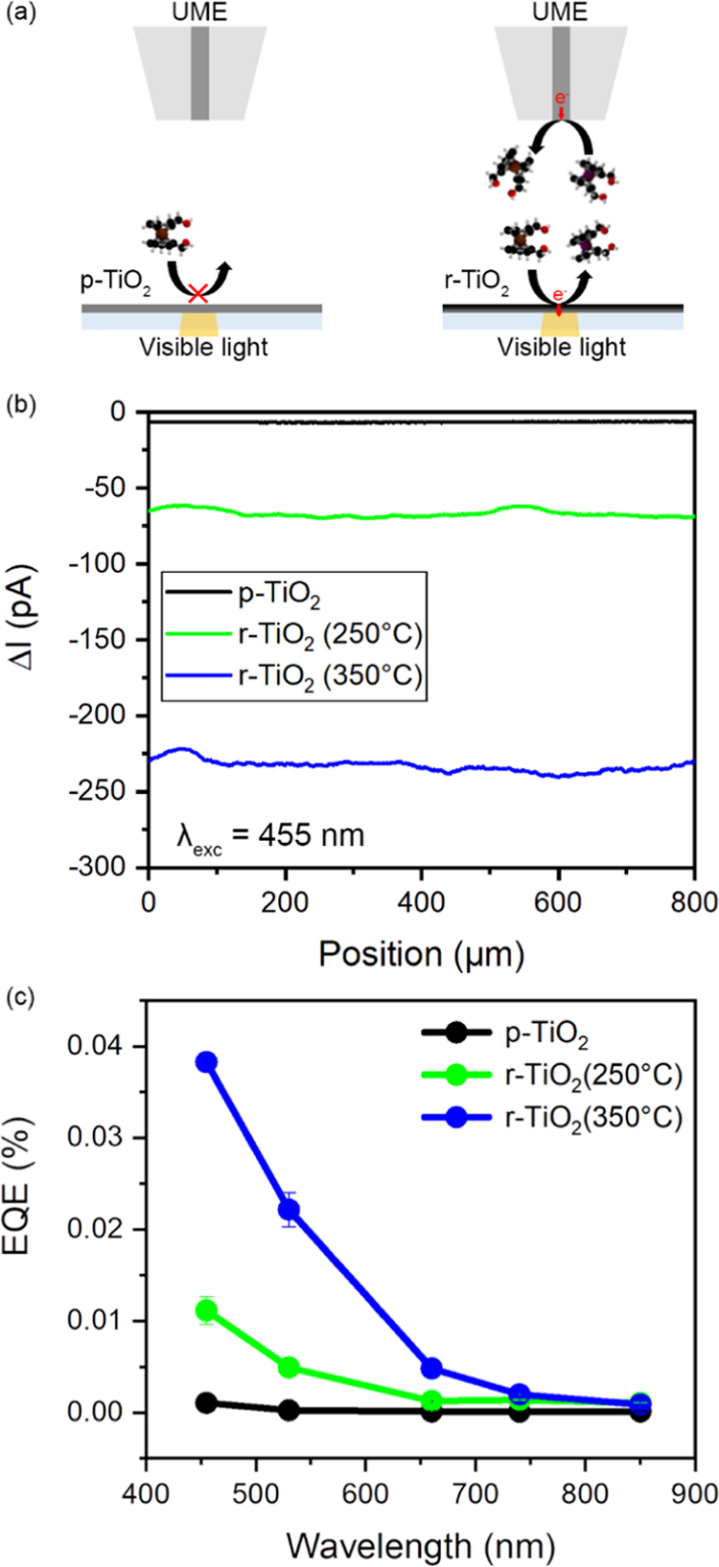
(a) Scheme illustrating a generic pristine (p-TiO_2_) and reduced TiO_2_ (r-TiO_2_) surface investigation by SPECM. (b) Line scan profile of the photoactivity at 455 nm (∼5.6 μW) and (c) EQE as a function of the excitation wavelength for the annealed TiO_2_ films on ITO (black, p-TiO_2_; green, r-TiO_2_ (250 °C); blue, r-TiO_2_ (350 °C)).

Next, we investigated the different TiO_2_ films by SPECM to probe the local photoactivity enhancement from the reduction process. The SPECM probe detects and quantifies the oxidized species photoproduced by the TiO_2_–ITO films (Figure 2a). The same conditions were applied to measure Δ*I*_p-TiO_2__, Δ*I*_r-TiO_2_ (250 °C)_, and Δ*I*_r-TiO_2_ (350 °C)_: the probe (*r*_T_ = 5 μm and RG = 5) was positioned at d = 20 μm, and the TiO_2_ films were scanned by an LED (455 nm) at 50 μm/s (Figure 2b). We employed an excitation source with a photon energy of about 2.7 eV (∼455 nm) to selectively probe the visible light photoactivity induced by the electronic states due to defects in anatase TiO_2_, which, in the pristine form, has a band gap energy of about 3.2 eV (∼390 nm).^51^ As expected, using 455 nm excitation, the measured Δ*I*_p-TiO_2__ was very low (−6.5 pA). In contrast, when considering the reduced samples, we detected Δ*I* values of −65 and −230 pA for r-TiO_2_ (250 °C) and r-TiO_2_ (350 °C), respectively. Notably, these differential currents corresponded to 10- and 35-fold enhancements of Δ*I*_455 nm_ with respect to p-TiO_2_. In order to gain insights into this behavior, we quantified the wavelength-dependent photooxidation efficiency of the different TiO_2_ films by retrieving the EQE. This was possible by calculating the substrate current thanks to a diffusion model, which enables the calculation of the collection efficiency (ratio between tip and substrate currents).^29^ The obtained EQE corresponds to the generation rate of the photogenerated holes reacting at the interface (TiO_2_–electrolyte). The EQE curves (Figure 2c) of the three investigated samples showed straightforwardly the effect of the reduction process on TiO_2_ activity in the visible light region. While p-TiO_2_ showed negligible photoactivity in the whole wavelength range, r-TiO_2_ (250 °C) and r-TiO_2_ (350 °C) showed significant photoactivity, which, for both samples, was higher at 455 nm and decreased at longer wavelengths (530, 660, 740, and 850 nm). Notably, a 90-fold enhancement in the EQE was observed at 530 nm between p-TiO_2_ and r-TiO_2_ (350 °C). A significant increase in photoactivity was also observed for longer wavelengths. For example, at 740 nm we observed an EQE of 10^–4^, 1.4 × 10^–3^, and 2 × 10^–3^ % for p-TiO_2_, r-TiO_2_ (250 °C), and r-TiO_2_ (350 °C), respectively. The sample reduced at a higher temperature, r-TiO_2_ (350 °C), showed higher photoactivity at all wavelengths in comparison to r-TiO_2_ (250 °C), suggesting the formation of a higher number of intragap states with various energy positions that participate in the photocatalytic process.

This investigation demonstrated the presence of intragap states due to defects’ formation in TiO_2_ ultrathin films and enabling broader light harvesting and photoactivity. These results are in agreement with previous reports on reduced TiO_2_ photoactivity enhancement in the visible light region.^8,41,42^ Moreover, our method evidenced, through a fast and facile strategy, the effect of the ultrathin films’ surface modification on the photogenerated carriers in the experimental conditions for a photocatalytic study where other commonly used characterization techniques can be blind in detecting structural and electronic modification due to a very low volume concentration of defects (e.g., Raman and PL spectroscopy).

### Mapping the Defect States Impact on Plasmonic Photocatalysis

**Figure 3:**
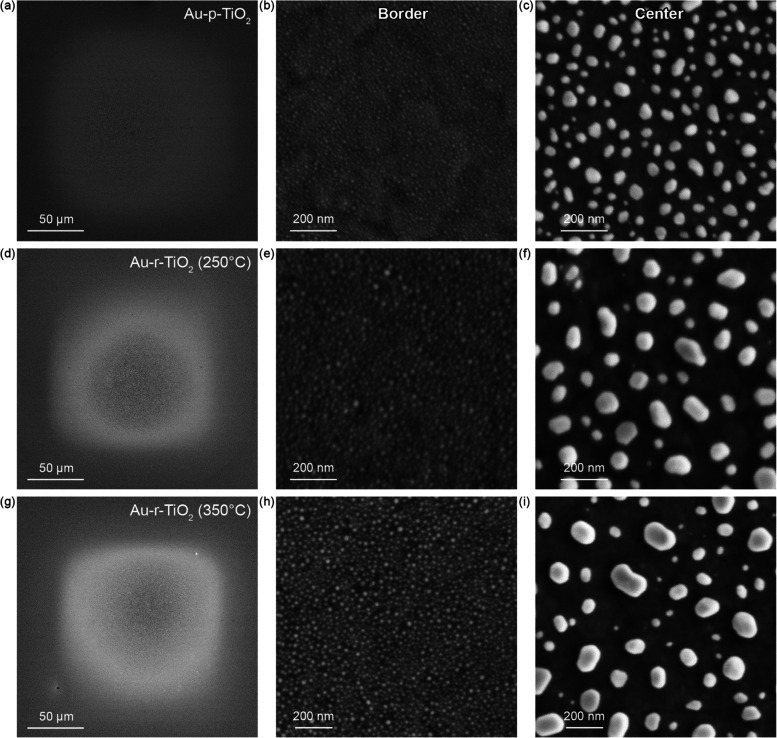
SEM images of different Au-TiO_2_ investigated showing (a–c) the entire Au NP array, (b–h) the border of the Au NP array (60 μm from the center), and (c–i) the center of the Au NP array for (a–c) Au-p-TiO_2_, (d–f) Au-r-TiO_2_ (250 °C), and (g–i) Au-r-TiO_2_ (350 °C).

After the detection of the visible light activity of the bare TiO_2_ films, we focused on investigating the effect of the reduction treatments on the plasmonic photoactivity of Au NP arrays deposited on TiO_2_. The Au-TiO_2_–ITO films were prepared by sputtering Au (10 nm thick) onto previously deposited TiO_2_ (30 nm thick) on ITO. A patterned mask created an ordered grid of Au squares (100 μm side; 400 μm interdistance) that enabled multiple sample investigations in one experiment, thus evaluating instantaneously the reproducibility of the collected data. The squares’ perimeter induced the formation of a gradient in the Au NP size distribution from the border to the center (Figure 3); less neighboring gold is available at the border of the square to form Au NPs during the dewetting process. To confirm the size distribution gradient, SEM images were recorded both in the squares’ border (Figure 3b,e,h) and in the center (Figure 3c,f,i,).

**Figure 4:**
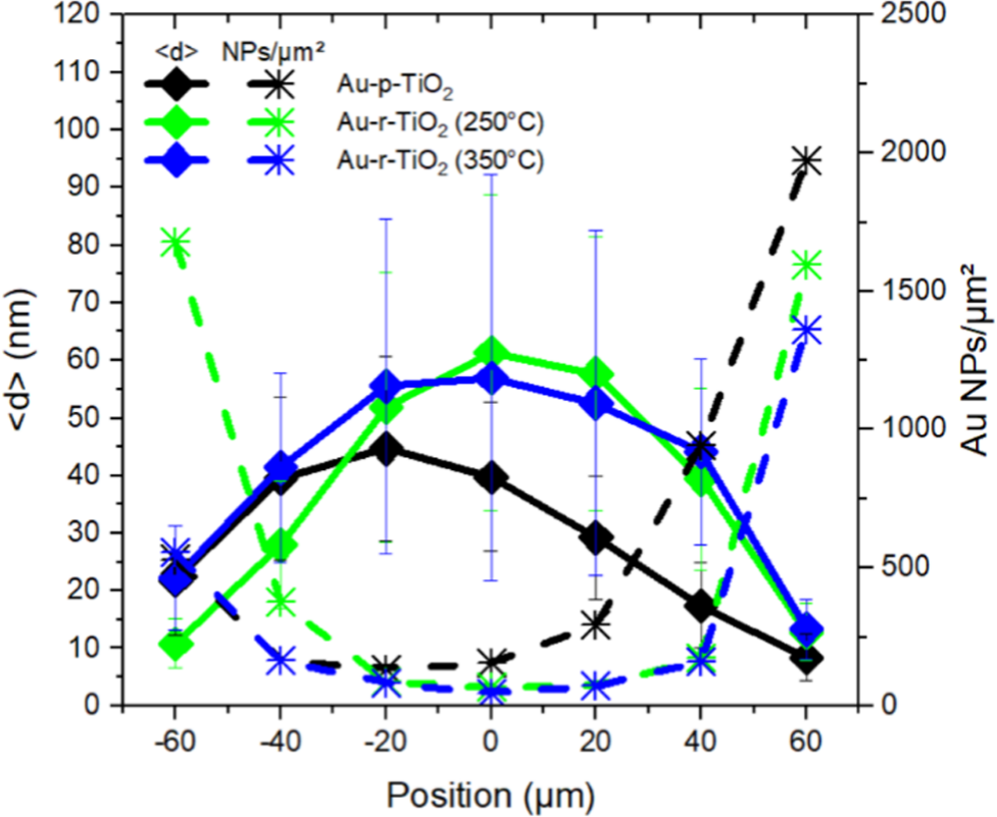
Mean diameter (<*d*>, solid line and filled diamond) with the standard deviation (error bars) and NP density (Au NPs/μm^2^, dashed line and star) of the Au NP array on TiO_2_–ITO films (black, p-TiO_2_; green, r-TiO_2_(250 °C); blue, r-TiO_2_(350 °C)) function of the position inside the Au NP array with 0 μm being the center of the square.

The Au NP size distribution was calculated from different SEM images collected every 20 μm from each other from the center of the array, i.e., a position of 0 μm (Figure 4). The mean diameter (<*d*>) of the Au NPs increased from the edge (∼12 nm) to the center (∼50 nm) of the square, and consequently, their spatial density decreased from ∼1500 to ∼100 particles/μm^2^. The same trend was observed for all investigated squares.

The optical properties of the Au NP arrays from Au-p-TiO_2_ and Au-r-TiO_2_ (350 °C) samples (Figure S7), obtained by SSM,^29^ highlighted (i) similar optical properties for both Au NP arrays and (ii) the LSPR peak position shifting from 560 to 610 nm from the border (−60 μm) to the center (0 μm) of the arrays due to the Au NP size increase. Both the morphology and optical properties of the different investigated samples closely resembled each other, which ensured a fair discussion on the effect of the TiO_2_ surface state on the Au NP plasmonic activity.

**Figure 5:**
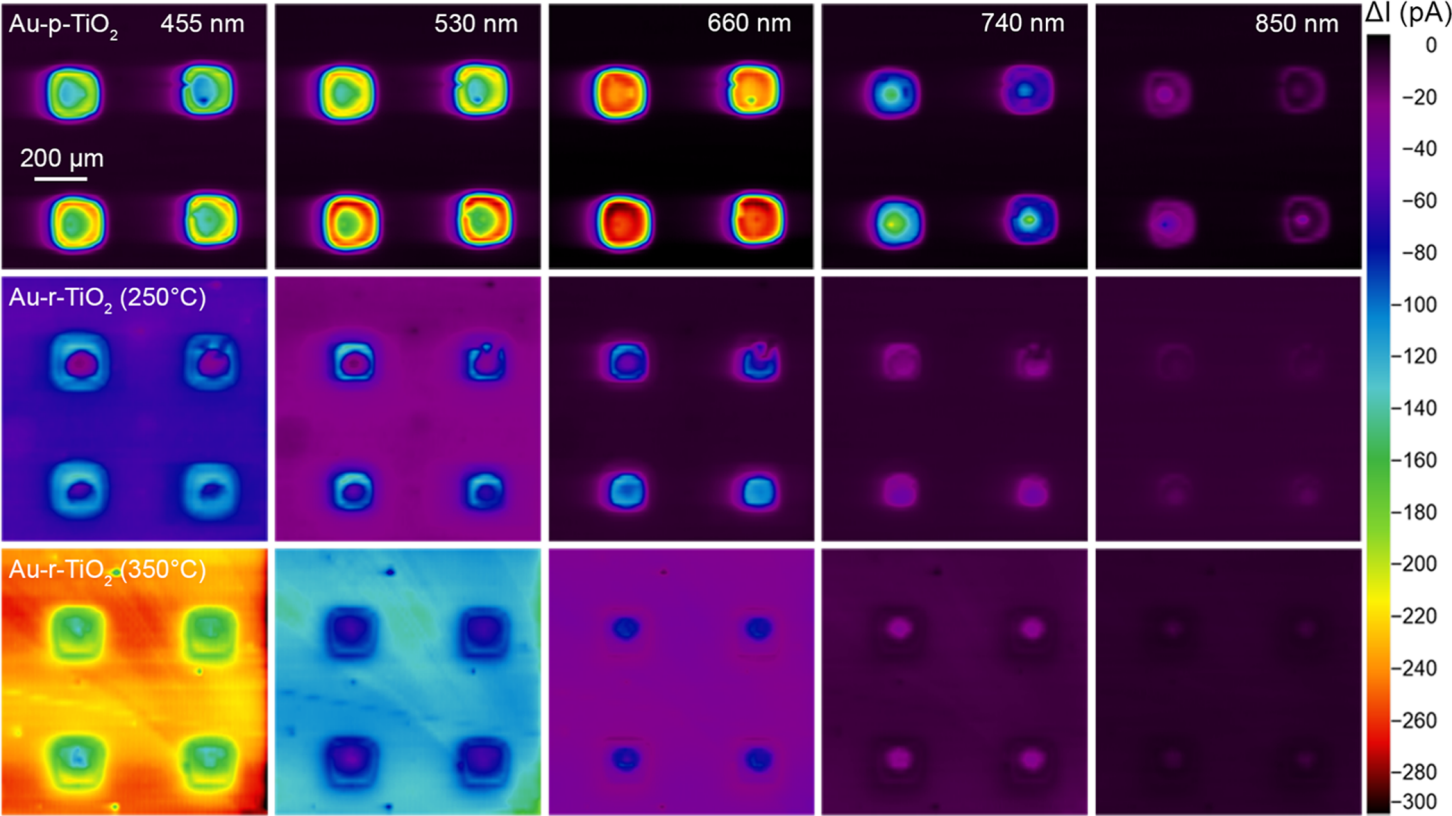
Photoactivity maps of 4 Au NP arrays from Au-p-TiO_2_, Au-r-TiO_2_ (250 °C), and Au-r-TiO_2_ (350 °C) excited at 455, 530, 660, 740, and 850 nm at 5.6, 4.7, 4.7, 4.2, and 5.1 μW, respectively. SPECM measurements were performed by SG-TC mode with a Pt probe (*r*_T_ = 5 μm, RG = 5), *d* = 20 μm, and v_scan_ = 50 μm/s in 1 mM FcDM and 0.1 M KCl. All of the maps have the same scale (1 mm^2^).

The two-dimensional mapping of multiple Au NP arrays was performed at different excitation wavelengths for pristine and reduced TiO_2_ films (Figure 5). Au-p-TiO_2_ photoactivity maps highlight higher currents at the border of the arrays, with the photoactivity increasing as |Δ*I*_455 nm_| < |Δ*I*_530 nm_| < |Δ*I*_660 nm_|. The maps measured with excitation at 455 and 530 nm evidenced a stark difference between the center and the border of the Au NP arrays as a consequence of a smaller Au NP size located at the array’s border (Figure 4) and in agreement with their optical properties (Figure S7). The activities observed at these wavelengths (455 and 530 nm) include the contribution of both plasmonic hot holes and *d*-holes, with the former generated mainly through a surface mechanism and the latter deriving from the whole Au NP volume.^52,53^

Interestingly, at 660 nm, where the surface plasmon excitation dominates the charge carrier generation mechanism,^52^ similar values both at the center and the border of the arrays were measured despite the increase of −log(*T*) values (−log(*T*)_660 nm_ = 0.06 and 0.18 at positions −60 and 0 μm, respectively) due to the bigger Au NPs. They feature a lower Au-TiO_2_ interface area (i.e., injection site for hot electrons) with respect to the smaller Au NPs that characterize the border of the array, thus ensuring a lower charge separation at the Au-TiO_2_ junction. Therefore, a trade-off between optical properties and the interface area is responsible for the observed activities of the border and the center of the array at 660 nm. For higher wavelengths, the Au NPs exhibited lower reactivity (740 nm) to barely any (850 nm). The observed trend is expected considering the size dependency on the optical properties and the electromagnetic field enhancement: the absorption/scattering ratio and the hot charge carrier generation decrease function of the NP size.^54,55^ The size of the NPs significantly affects the density of charge injected across the ϕ_B_, thus the system plasmon-driven photoactivity.

In the case of Au-r-TiO_2_, we observed an activity decrease in the area containing the plasmonic Au NP array at all investigated wavelengths, in agreement with the H_2_ treatment temperature (|Δ*I*|_Au-*p*-TiO_2__ > |Δ*I*|_Au-*r*-TiO_2_ (250 °C)_ > |Δ*I*|_Au-*r*-TiO_2_ (350 °C)_). In contrast, the photoactivity outside the plasmonic squares, i.e., where only TiO_2_ is present, increased at all wavelengths following an opposite trend (|Δ*I*|_Au-*r*-TiO_2_ (350 °C)_ > |Δ*I*|_Au-*r*-TiO_2_ (250 °C)_ > |Δ*I*|_Au-*p*-TiO_2__).

**Figure 6:**
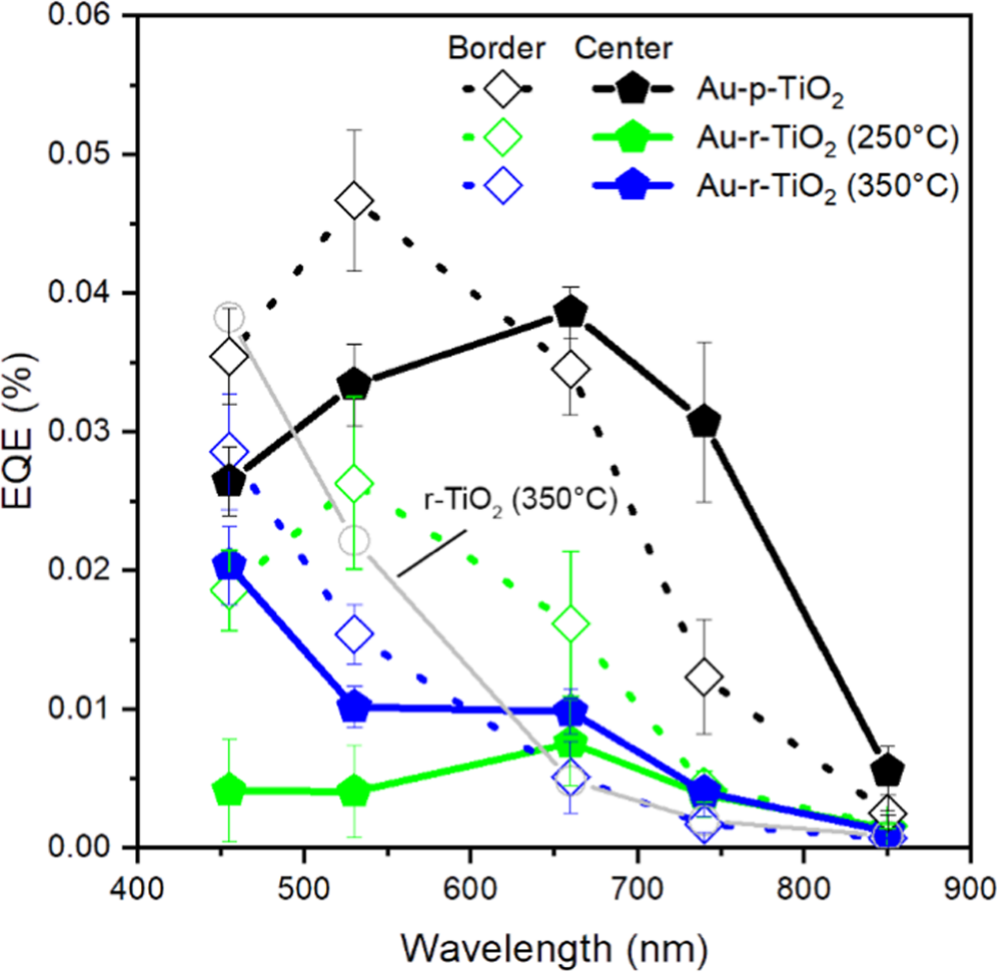
EQE function of the excitation wavelength at the border (dotted line and filled diamond) and the center (solid line and filled pentagon) of the Au NP array on TiO_2_–ITO films (black, p-TiO_2_; green, r-TiO_2_ (250 °C); blue, r-TiO_2_ (350 °C)) and r-TiO_2_ (350 °C) (gray).

As expected from the population distribution and optical properties, the EQE evolved from the border to the center of the square due to the increase in the Au NP <*d*> (Figure 6).^29,56,57^ Indeed, the wavelength providing the highest EQE for Au-p-TiO_2_ shifted due to the increase in <*d*> and was observed at 530 and 660 nm for the border and the center of the arrays, respectively. These results are in agreement with the LSPR peak shift observed from the optical properties (Figure S7). In contrast, in the case of the reduced samples, we observed different behaviors. In the center of the Au NP arrays, the EQE of Au-r-TiO_2_ (250 °C) and Au-r-TiO_2_ (350 °C) peaked at 660 nm due to the plasmonic activity but with a 5- and 4-times decrease compared to Au-p-TiO_2_, respectively. At lower wavelengths, the EQE of Au-r-TiO_2_ (250 °C) is very low (∼7-fold lower than Au-p-TiO_2_), while that of Au-r-TiO_2_ (350 °C) shows higher values than Au-r-TiO_2_ (250 °C). The higher EQE of Au-r-TiO_2_ (350 °C) compared to Au-r-TiO_2_ (250 °C) is due to the increased contribution from the TiO_2_ substrate, in agreement with the EQE of r-TiO_2_ (350 °C) (EQE_455 nm_ = 0.038%, 0.004%, and 0.020%; EQE_530 nm_ = 0.022%, 0.004%, and 0.010%; EQE_660 nm_ = 0.005%, 0.008%, and 0.010% for r-TiO_2_ (350 °C), center-Au-r-TiO_2_ (250 °C) and center-Au-r-TiO_2_ (350 °C), respectively). When we consider the border of the array, the EQE curve of Au-r-TiO_2_ (250 °C) still shows the characteristic plasmonic activity peak (∼2-fold lower than that of Au-p-TiO_2_). In contrast, the EQE of Au-r-TiO_2_ (350 °C) did not reveal any plasmonic peak, featuring a higher activity at 455 nm and a rapid decrease at longer wavelengths closely resembling the r-TiO_2_ (350 °C) trend (Figure 6). As evidenced in Figure 2c, the EQE of r-TiO_2_ (250 °C) is ∼4 times lower than that of r-TiO_2_ (350 °C). Thus, we can extrapolate that the reduction process at 250 °C induces a 4-times lower quantity of defects than that at 350 °C. However, the population distributions of Au NPs remained similar (Figure 4), which explains the preserved feature of the plasmonic activity for Au-r-TiO_2_ (250 °C).

Our results suggest that the plasmonic quenching is related to the charge recombination between the Au NPs and r-TiO_2_. Indeed, at shorter wavelengths (455 and 530 nm), a total quenching of the plasmonic activity is observed due to the significant charge generation in r-TiO_2_ (EQE_455 nm_ = 0.038%, 0.029%, and 0.020%; EQE_530 nm_ = 0.022%, 0.015%, and 0.010% for r-TiO_2_ (350 °C) and border- and center-Au-r-TiO_2_ (350 °C), respectively). In contrast, at a longer wavelength (660 nm), the plasmonic activity is only decreased, i.e., the plasmonic peak is still visible, due to lesser charge generation in TiO_2_ (EQE_660 nm_ = 0.005%, 0.005%, and 0.010% for r-TiO_2_ (350 °C) and border- and center-Au-r-TiO_2_ (350 °C), respectively). These results are in accordance with previous reports comparing Au NPs on pristine and defective TiO_2_.^12,15^

Ultimately, we ascribe these observations to the presence of interfacial defect–electron traps acting as recombination centers and enabling a pathway for electrons generated within TiO_2_ to recombine with the hot holes present in Au NPs, thus decreasing the overall photoreactivity of the latter.^12,15^ Above a certain threshold of defect concentrations, the plasmonic quenching effect is observed due to the significant quantity of charges generated in TiO_2_.

**Figure 7:**
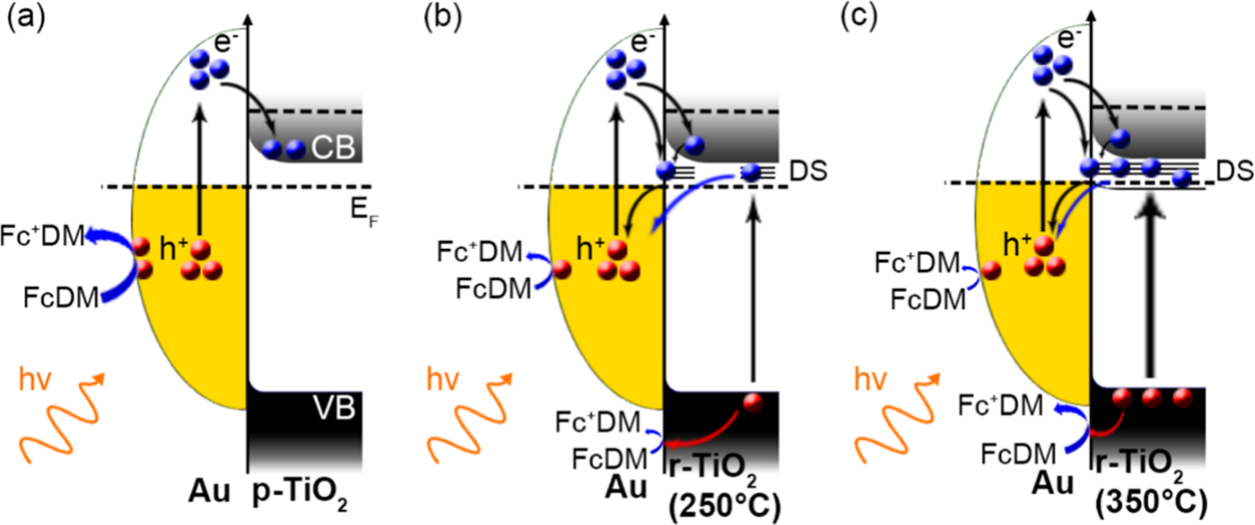
Schemes depicting the charge recombination scenarios under visible light excitation. (a) Au NPs on p-TiO_2_, (b) Au NPs on r-TiO_2_ (250 °C) with a low amount of DSs, and (c) Au NPs on r-TiO_2_ (350 °C) with a high amount of DSs.

These considerations can be summarized in three possible scenarios, representing recombination processes occurring in the photocatalysts (Figure 7).

In the first case, Au-p-TiO_2_ shows the expected photoactivity behavior and can be considered as the reference scenario where, at any wavelengths, the excited electronic transitions belong only to Au NPs, and the hot holes (either d or plasmonic in nature) do not undergo recombination with charges photogenerated in TiO_2_ as it ideally contains no defects and, thus, no intragap interfacial states (Figure 7a).

In the case of the reduced samples, we observed that the TiO_2_ defects/electronic states introduced at the Au-TiO_2_ interface acted as follows: (i) increasing the charge generation (photoactivity) upon visible light excitation in reduced TiO_2_, (ii) providing a recombination pathway for hot charge carriers, and (iii) introducing leaking pathways for electrons from TiO_2_ to the Au NPs. Finally, the increase in H_2_ annealing temperature introduces more surface/interfacial defects, increasing the TiO_2_ photoactivity while decreasing the plasmon-driven activity through the charge generated in TiO_2_ leaking into Au NPs and the higher recombination rate for hot charge carriers (Figure 7b,c).

## Conclusions

Based on SPECM investigations, we realized fast and simple *in situ* mapping of the photoactivity of hybrid nanostructures (Au-TiO_2_). The investigation of different TiO_2_ ultrathin films revealed the effect of H_2_ annealing on the chemical products generated upon visible light excitation. Our results evidenced the significant enhancement (EQE 30- to 90-fold higher in the blue-green region) on the charge carrier generation due to induced surface defects on ultrathin TiO_2_ films even under a mild reduction process (350 °C, 1 h with 5% H_2_). The two-dimensional mapping of Au NP arrays on the different TiO_2_ films evidenced that the surface/interfacial defects drastically quenched the plasmon*-*driven photoactivity due to intragap states in TiO_2_ acting as hot electron traps and leaky pathways for electrons from TiO_2_ to travel to Au NPs. Moreover, this effect showed a dependency on the Au NP size distribution and the concentration of defects, also displaying wavelength-dependent generation and recombination processes. The versatility of SPECM facilitates the analysis of the *in situ*/*operando* chemical photoactivity of engineered surfaces on plasmonic-semiconductor nanostructures. The resulting information fundamental insights will improve the comprehension of these systems, thus supporting the design of efficient photocatalysts.

## Supporting Information Available

Containing Raman, PL spectroscopy, XPS, and additional SPECM data (PDF)
