# Supplementary material for: Interfacial States in Au/Reduced TiO2 Plasmonic Photocatalysts Quench Hot-Carrier Photoactivity
Source: J Phys Chem C Nanomater Interfaces. 2023 Aug 7;127(32):15861–70. doi: 10.1021/acs.jpcc.3c04176 (PMC10441571; doi:10.1021/acs.jpcc.3c04176)
Supplement: Supplementary file 1 — jp3c04176_si_001.pdf [file jp3c04176_si_001.pdf]

Supplementary information of "Interfacial States in Au/Reduced-TiO_2 Plasmonic Photocatalysts Quench Hot Carriers Photoactivity"

Olivier Henrotte,[1] Štěpán Kment,[1,2] and Alberto Naldoni[1,3*]

[1] Czech Advanced Technology and Research Institute, Regional Centre of Advanced Technologies and Materials Department, Palacký University Olomouc, Šlechtitelů 27, Olomouc 78371, Czech Republic

[2] CEET, Nanotechnology Centre, VŠB-Technical University of Ostrava, 17. Listopadu 2172/15, Ostrava-Poruba 708 00, Czech Republic

[3] Department of Chemistry and NIS Centre, University of Turin, Turin 10125, Italy

E-mail: alberto.naldoni@unito.it

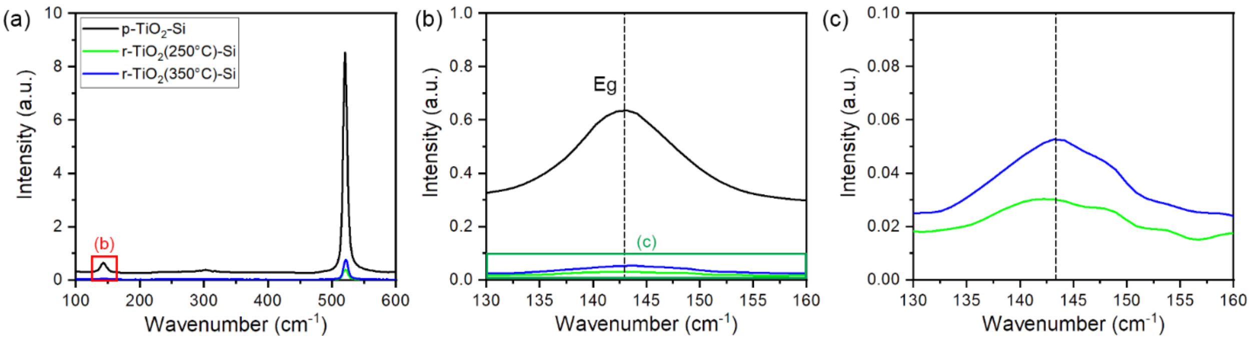

Figure S1. (a-c) Raman spectra of the annealed TiO_2 films grown on silicon wafer (black, p-TiO_2-Si; green, r-TiO_2(250°C)-Si; blue, r-TiO_2(350°C)-Si) with (b) and (c) corresponding to the zoomed-in area framed in the previous panel. The black dotted line highlights the E_g peak from TiO_2 in (b) and (c).

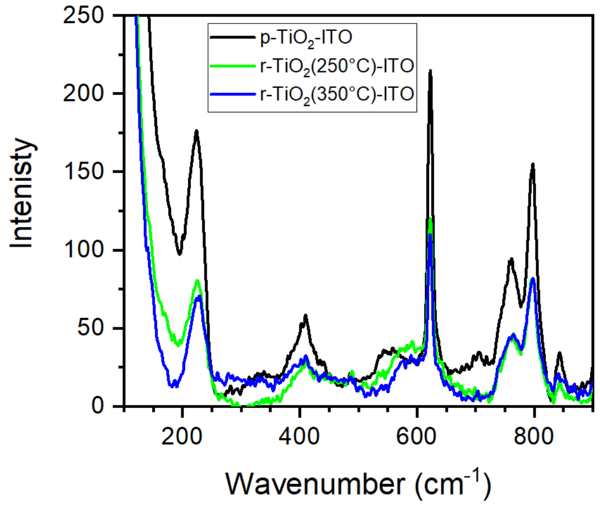

Figure S2. Raman spectra of the annealed TiO_2 films grown on ITO (black, p-TiO_2-ITO; green, r-TiO_2(250°C)-ITO; blue, r-TiO_2(350°C)-ITO).

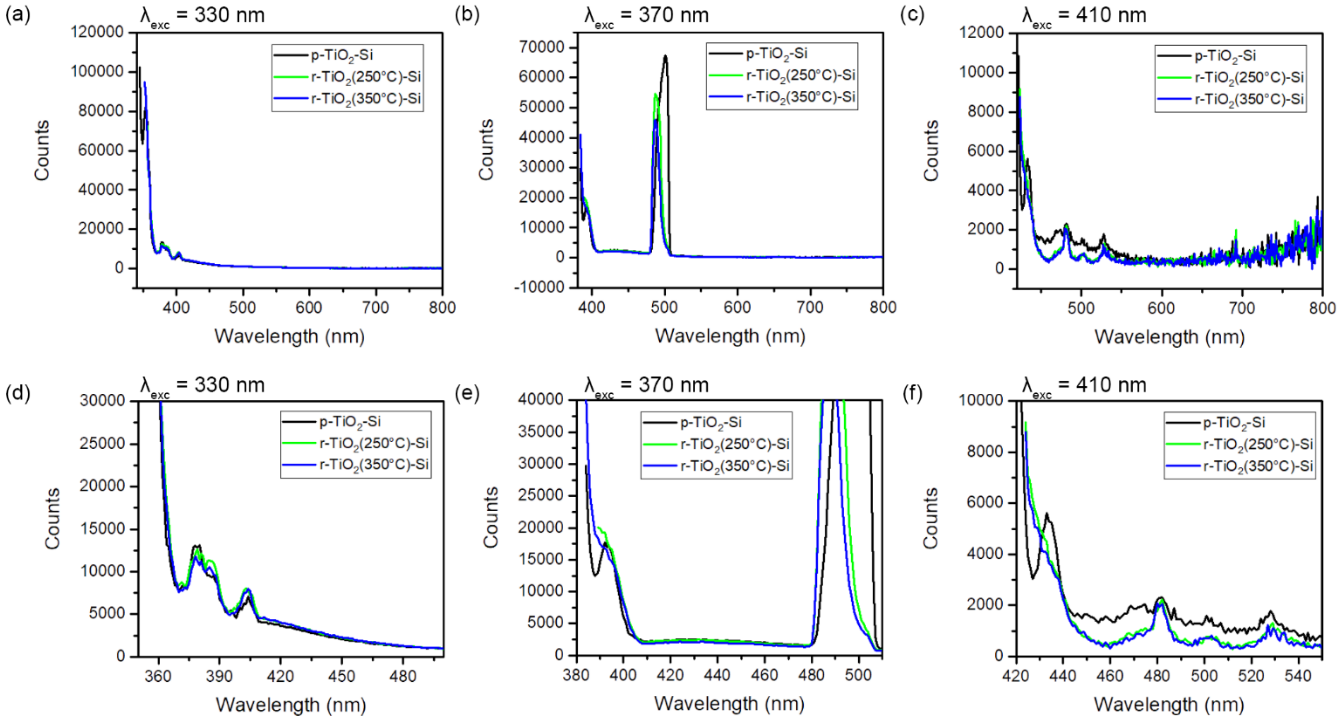

Figure S3. Photoluminescence spectra of the TiO_2 annealed (black, p-TiO_2-Si; green, r-TiO_2(250°C)-Si; blue, r-TiO_2(350°C)-Si) with different excitation wavelengths: (a,d) 330 nm, (b,e) 370 nm, and (c,f) 410 nm.

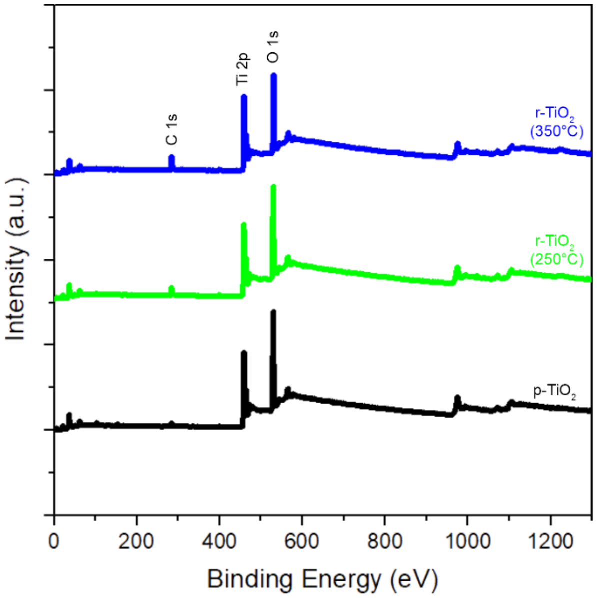

Figure S4. XPS surveys of the annealed TiO_2 films grown on ITO (black, p-TiO_2; green, r-TiO_2(250°C); blue, r-TiO_2(350°C)).

Table S1. Values corresponding to the atomic percentage from the surveys in Figure S4 for Ti 2p, O 1s and C 1s peaks.

**Sample**	**p-TiO_2**	**r-TiO_2 (250°C)**	**r-TiO_2 (350°C)**
**Ti 2p**	22.14	21.12	19.01
**O 1s**	68.37	61.26	54.28
**C 1s**	9.49	17.62	26.71

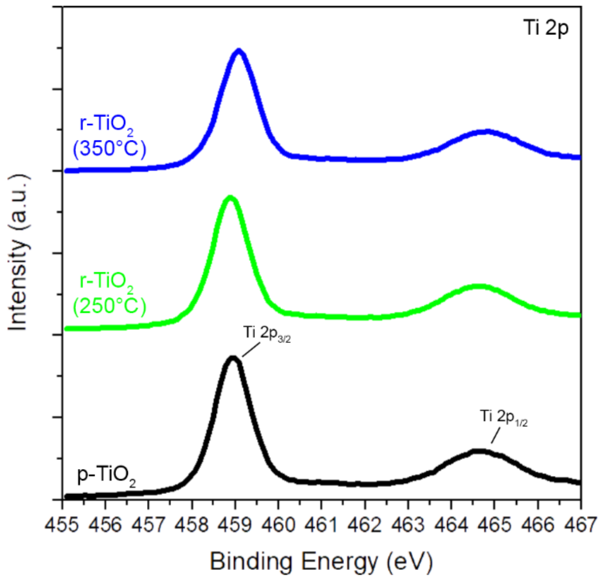

Figure S5. XPS Ti 2p high resolution spectra of the annealed TiO_2 films grown on ITO (black, p-TiO_2; green, r-TiO_2(250°C); blue, r-TiO_2(350°C)).

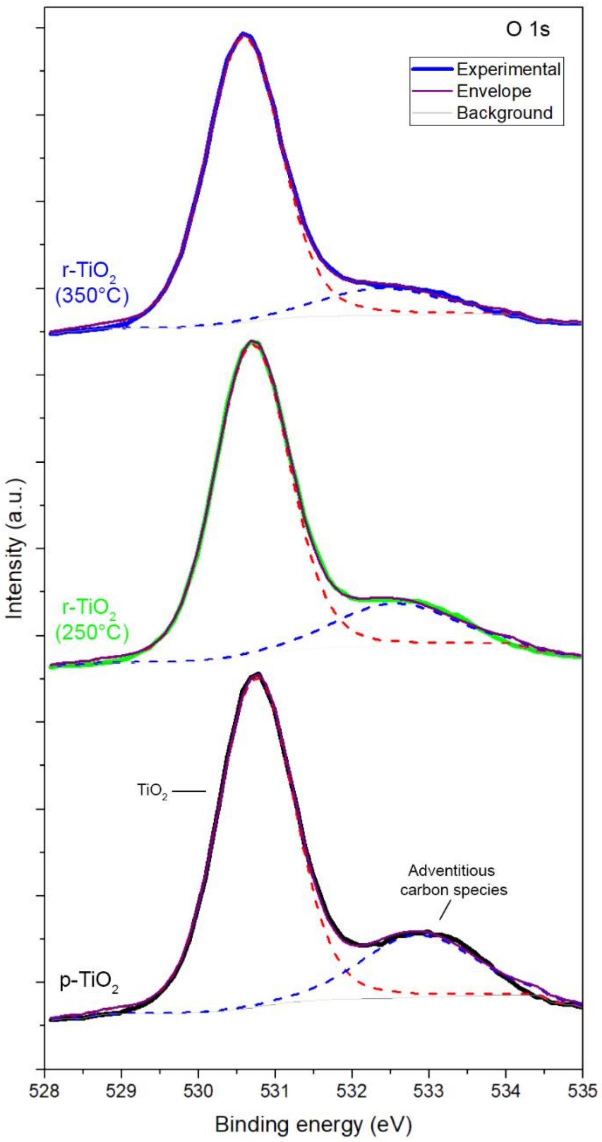

Figure S6. XPS O 1s high resolution spectra of the annealed TiO_2 films grown on ITO (black, p-TiO_2; green, r-TiO_2(250°C); blue, r-TiO_2(350°C)).

Table S2. Values corresponding to the different parameters of the deconvoluted O 1s peaks presented in Figure S6.

**Sample**	**p-TiO_2**		**r-TiO_2 (250°C)**		**r-TiO_2 (350°C)**	
**Peak attribution**	TiO_2	Adv. C	TiO_2	Adv. C	TiO_2	Adv. C
**Peak position (eV)**	530.7	532.8	530.7	532.5	530.6	532.4
**FWHM (eV)**	1.17	1.74	1.15	1.78	1.15	1.83
**Concentration (%)**	78.07	21.93	82.37	17.63	87.02	12.98

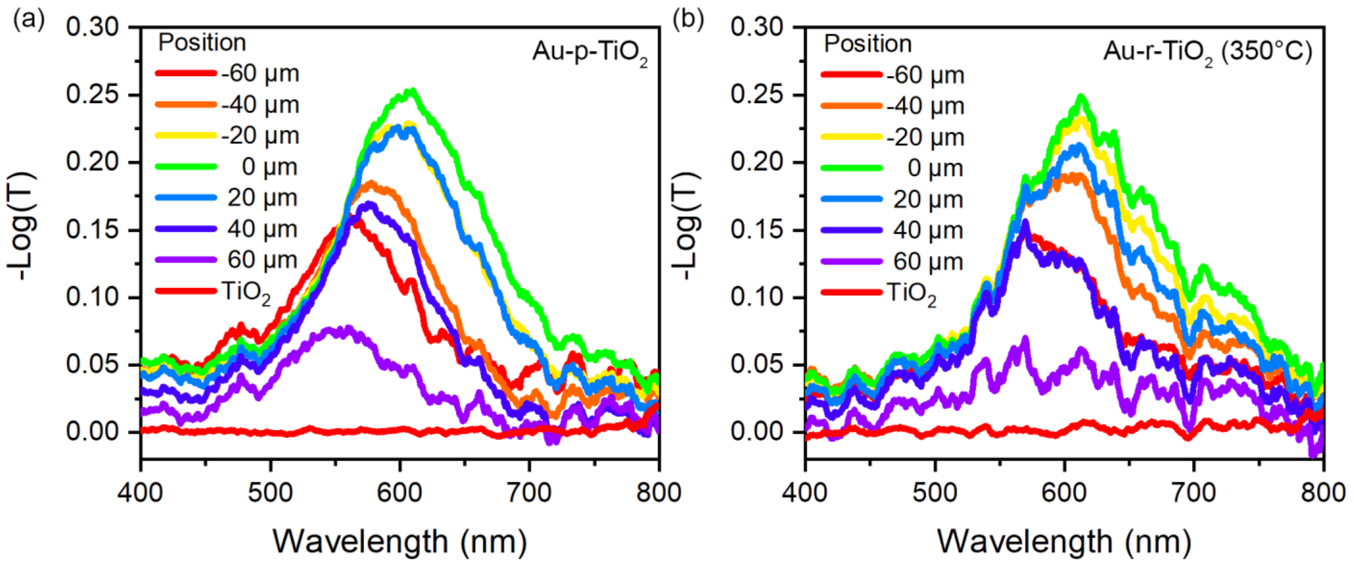

Figure S7. Optical measurements of Au NP arrays on (a) p-TiO_2 and (b) r-TiO_2 (350°C) taken at different positions inside the arrays (0 µm = center of the array).
